# Treatment outcomes of mucosal melanoma of head and neck: Efficacy of immune checkpoint inhibitors for advanced disease

**DOI:** 10.3389/fsurg.2022.1032626

**Published:** 2022-12-26

**Authors:** Shusuke Ohshima, Yushi Ueki, Yusuke Yokoyama, Takeshi Takahashi, Ryusuke Shodo, Keisuke Yamazaki, Ryuichi Okabe, Hiroshi Matsuyama, Takafumi Togashi, Sumiko Takatsuka, Tatsuya Takenouchi, Arata Horii

**Affiliations:** ^1^ Department of Otolaryngology Head and Neck Surgery, Niigata University Graduate School of Medical and Dental Sciences, Niigata, Japan; ^2^ Division of Otorhinolaryngology, Nagaoka Red Cross Hospital, Niigata, Japan; ^3^ Division of Otorhinolaryngology, Niigata City General Hospital, Niigata, Japan; ^4^ Division of Head and Neck Surgery, Niigata Cancer Center Hospital, Niigata, Japan; ^5^ Division of Dermatology, Niigata Cancer Center Hospital, Niigata, Japan

**Keywords:** Head and neck mucosal melanoma, Advanced disease, Immune checkpoint inhibitor, Immune-related adverse events, Overall Response Rate (ORR)

## Abstract

**Background:**

Head and neck mucosal melanoma (HNMM) is a rare and aggressive subtype of melanoma. HNMM often develops as a recurrent or metastatic disease, and its prognosis is worse than that of cutaneous melanoma. Recent large-scale clinical studies have reported favorable outcomes with immune checkpoint inhibitors (ICIs) for melanoma. However, these clinical trials included only a small number of HNMM cases. This study aimed to estimate treatment outcomes and prognostic predictors of ICIs for advanced HNMM.

**Methods:**

Cases of advanced HNMM, defined as unresectable or metastatic HNMM at the initial diagnosis (five patients) or development of recurrent/metastatic HNMM after initial treatment (27 patients), were included in this study. Survival analysis and a search for prognostic factors were performed for these 32 patients. Furthermore, the detailed clinical course of patients who received ICI treatment was investigated.

**Results:**

The median overall survival (OS) of 32 patients with advanced HNMM was 25.3 months. The estimated 1-, 3-, and 5-year OS rates were 68.4%, 42.8%, and 34.3%, respectively. Fourteen patients (43.7%) received ICIs, whereas 18 (56.3%) did not. Univariate analysis showed that ICI treatment was the only factor associated with a better 1-year OS. Patients who received ICI treatment had significantly longer OS (median OS: not reached, 1-year OS: 85.7%) than those who did not (median OS: 11.3 months, 1-year OS: 54.5%). The overall response and disease control rates of patients who received ICI treatment were 50% and 64.3%, respectively. Patients who achieved complete response (CR) or partial response (PR) to ICI treatment survived significantly longer (1-year OS: 100%) than those who did not (1-year OS: 71.4%). Among the five patients who discontinued ICI treatment due to severe immune-related adverse events (irAEs), four did not receive salvage treatments but showed durable treatment effects and survived for 9.8–54.2 months at the end of the follow-up period.

**Conclusions:**

ICI treatment achieved a favorable OS for advanced HNMM. CR/PR to ICI treatment and discontinuation owing to severe irAEs were favorable predictors of OS.

## Introduction

Mucosal melanoma (MM) is a rare melanoma subtype that accounts for only 1.3% of all melanoma cases in the United States (1). MM is relatively more common in Asian countries (8%–22.6% of all melanomas) (2, 3), possibly because of the low incidence of cutaneous melanoma (CM). Among MM, 34%–40% arise from the head and neck regions (4, 5). The 5-year-overall survival (OS) of head and neck MM (HNMM) is less than 30% (6, 7), while that for CM is more than 80% (8), indicating that HNMM has a poorer prognosis than CM. Based on the aggressive nature of MM, all HNMM cases are classified as stage III or higher according to the TNM classification (9). The sinonasal and oral cavities are the two most common primary sites of HNMM. Since HNMM shows only non-specific symptoms in the sinonasal and oral cavities, such as epistaxis, nasal obstruction, or discomfort of the throat (10) at the initial stage, most patients are diagnosed only at the advanced stage, making disease management difficult. Furthermore, dermoscopy and confocal microscopy could be used for diagnosing oral MM as for CM and genitourinary MM (11–14), whereas it would be difficult to diagnose sinonasal MM by these devices. Given that early diagnosis of HNMM is often difficult, the development of effective treatment strategies for advanced melanoma is desirable.

Recent advances in immune checkpoint inhibitors (ICIs) have dramatically changed the treatment strategies for melanoma (15–18). In particular, anti-programmed death-1 antibodies, such as nivolumab and pembrolizumab, have significantly prolonged the survival time of patients with advanced melanoma in comparison with dacarbazine, the standard cytotoxic agent for advanced melanoma, and ipilimumab, a fully human monoclonal antibody against CTL antigen 4 (15, 17). However, patients enrolled in this phase III trial mostly had CM, and the proportion of MM patients was very small: 29 MM (3.2%) and 766 CM (84.5%) patients out of 906 melanoma patients (18). Moreover, no report to date has exclusively focused on the treatment outcome of ICI therapy for HNMM; previous reports have included subtypes other than HNMM (19–21). Therefore, this study aimed to examine the treatment outcomes of ICIs in patients with advanced HNMM who had unresectable and/or metastatic disease at the initial diagnosis or who developed recurrent and/or metastatic disease after the initial treatment.

## Materials and methods

This study was retrospective, multicenter, non-randomized study to evaluate the treatment outcomes of patients with advanced HNMM. This study was approved by the Institutional Review Board of Niigata University Medical and Dental Hospital (IRB No. 2019-0398).

### Patients

**Figure 1:**
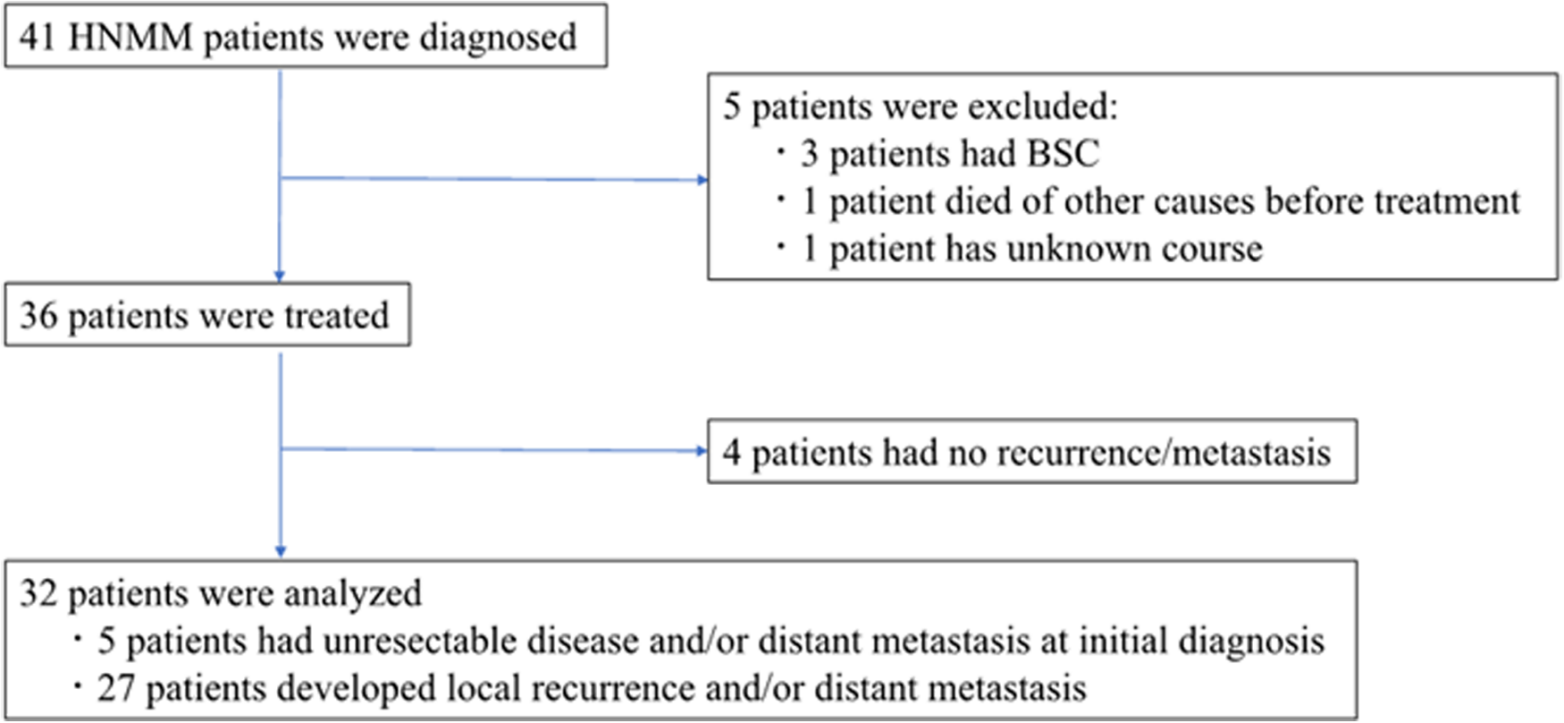
Flow diagram of study participants. Of the 36 HNMM patients who received any treatment, we excluded four patients who survived without recurrent/metastatic disease. The remaining 32 patients were analyzed: 5 patients had an unresectable disease and/or distant metastasis at the initial diagnosis, while 27 developed local recurrence and/or distant metastasis after the initial treatment.

The flow diagram of the study participants is shown in Figure 1. The inclusion criteria were as follows: previously untreated patients presenting with histologically proven MM between January 2001 and August 2021 at our institutions and showing advanced HNMM, defined as unresectable and/or metastatic disease at initial diagnosis or recurrent/metastatic disease after definitive treatments (19–21). A total of 41 patients were diagnosed with HNMM. Of the 36 patients who received definitive treatment, four who survived without recurrent/metastatic disease did not meet the criteria for advanced disease and were excluded from the analyses. Thus, a total of 32 patients were analyzed: five patients had an unresectable disease and/or distant metastasis at the initial diagnosis, while 27 developed local recurrence and/or distant metastasis after the initial treatment. Regarding disease status, 18 patients (56.3%) had unresectable primary tumors or local recurrence, whereas 14 (43.7%) showed distant metastasis alone (Table 1).

**Table 1:** Background comparison between ICI and non-ICI groups in patients with advanced HNMM.

Characteristic	ICI, *n* = 14 (%)	non-ICI, *n* = 18 (%)	*p*-value
**Age**			
median (range), years	72 (25–85)	77 (38–88)	0.403
**Sex**			
Male	7 (50)	11 (61.1)	0.721
Female	7 (50)	7 (38.9)	
**Primary site**			
Sinonasal cavity	11 (78.6)	15 (83.3)	1
other	3 (21.4)	3 (16.7)	
**Disease status**			
Unresectable/Local recurrence	7 (50)	11 (61.1)	0.721
Distant metastasis alone	7 (50)	7 (38.9)	
**BRAF status** ^a^			
Wild	14 (100)	–	–
Mutant	0 (0)	–	
**NRAS status^a^**			
Wild	10 (71.4)	–	–
Mutant	4 (28.6)	–	
**PD-L1 status^a^**			
<5% or Unknown	11 (78.6)	–	–
≥5%	3 (21.4)	–	

ICI, immune checkpoint inhibitor; NRAS, neuroblastoma RAS viral oncogene homolog; PD-L1, programmed death-ligand 1.

*Bold: statistically significant.

^a^ BRAF/NRAS/PD-L1 evaluation were performed only on patients who received ICI treatment.

Data on the following baseline characteristics were collected: age, sex, primary site, T classification, N classification, M classification, stage, treatment history, v-Raf murine sarcoma viral oncogene homolog B1 (BRAF)/neuroblastoma RAS viral oncogene homolog (NRAS) mutational status, and programmed death-ligand 1(PD-L1) expression.

### Treatment

The treatment strategy for HNMM at our institution has changed since the medical insurance approval of ICI in Japan in 2014. Before 2013, radical resection plus postoperative radiotherapy (RT) or heavy ion beams was performed as an initial treatment, and chemotherapy with dacarbazine was applied to recurrent or metastatic disease. Since 2014, ICI has been the key-drug in the treatment for recurrent/metastatic disease (advanced HNMM).

### Efficacy and toxicity assessments

OS was defined as the time from the initiation of treatment for advanced disease to the date of death from any cause. The survival time was estimated from the start of the initial treatment for five patients who were diagnosed with advanced HNMM at the initial diagnosis, while the survival time of patients who were diagnosed with advanced HNMM due to development of recurrence/metastasis after initial treatments was estimated from the start of the treatment for recurrent/metastasis. The overall response to ICI treatment was evaluated 8 weeks after the start of ICI treatment for advanced disease using the Response Evaluation Criteria in Solid Tumors criteria version 1.1 (22). Tumor response was categorized as complete response (CR), partial response (PR), stable disease (SD), or progressive disease (PD). The overall response rate (ORR) was defined as the proportion of CR plus PR, whereas the disease control rate was defined as the proportion of CR, PR, and SD. Immune-related adverse events (irAEs) were graded based on the National Cancer Institute Common Terminology Criteria for Adverse Events version 5.0.

### Evaluation of Pd-L1 positivity and BRAF/NRAS mutation

PD-L1 expression in surgical and biopsy specimens was evaluated by immunohistochemical testing (The Dako 28-8 pharmDx, Agilent Technologies/Dako, Carpinteria, CA) and grouped by expression levels, comprising levels of <5% and ≥5% in a minimum of 100 evaluated tumor cells, based on data from the Checkmate 067 trial (16).

BRAF/NRAS mutation analysis was performed using the THxID BRAF kit (bioMérieux Japan Ltd., Tokyo, Japan) and the MEBGEN RASKET™-B kit (Medical & Biological Laboratories Co. Ltd., Tokyo, Japan). We evaluated two types of BRAF exon 15 mutations (V600 E and V600K), 12 types of NRAS exon 2 (G12, G12C, G12R, G12D, G12V, G12A, G13S, G13C, G13R, G13D, G13V, and G13A), nine types of NRAS exon 3 (A59T, A59G, Q61K, Q61E, Q61L, Q61P, Q61R, Q61Ht, and Q61Hc), and five types of NRAS exon 4 (K117Nc, K117Nt, A146T, A146P, and A146V).

### Statistical analysis

Continuous variables were expressed as medians and ranges. Survival curves were constructed using the Kaplan–Meier method and compared using the log-rank test. All statistical analyses were two-sided, and the significance level was set at *p* < 0.05. All statistical analyses were performed using EZR (23).

## Results

### Patient characteristics

The characteristics at the diagnosis of advanced HNMM in Table 2. The median age was 72.5 years (range, 25–88 years), and 18 patients (56.3%) were male. The primary sites were the sinonasal cavity in 26 patients (81.3%) and other sites in six patients (18.7%). Eighteen patients (56.3%) had unresectable/local recurrence, whereas 14 patients (43.7%) had distant metastasis. As treatment for advanced disease, 14 patients (43.7%) received ICIs, whereas 18 patients (56.3%) did not receive ICI therapy. Among the patients who received ICI therapy, 12 were treated with nivolumab and one each with pembrolizumab and combination therapy of nivolumab and ipilimumab. Among the patients who did not receive ICI treatment, two patients underwent surgery, four received RT, one received chemotherapy, and 11 received the best supportive care.

**Table 2:** Characteristics at the diagnosis as advanced HNMM (*n* = 32).

Characteristic at the diagnosis as advanced HNMM	*n* = 32 (%)
**Age**	
median (range), years	72.5 (25–88)
**Sex**	
Male	18 (56.3)
Female	14 (43.7)
**Primary site**	
Sinonasal cavity	26 (81.3)
other	6 (18.7)
**Disease status**	
Unresectable/Local recurrence	18 (56.3)
Distant metastasis alone	14 (43.7)
**Treatments**	
With ICI	14 (43.7)
Without ICI	18 (56.3)
**BRAF status^a^ (*n* = 14)**	
Wild	14 (43.7)
Mutant	0 (0)
**NRAS status^a^ (*n* = 14)**	
Wild	10 (31.2)
Mutant	4 (12.5)
**PD-L1 status^b^ (*n* = 12)**	
<5%	9 (28.1)
≥5%	3 (9.4)

HNMM, head and neck mucosal melanoma; ICI, immune checkpoint inhibitor; BRAF, v-Raf murine sarcoma viral oncogene homolog B1; NRAS, neuroblastoma RAS viral oncogene homolog; PD-L1, programmed death-ligand 1

^a^ BRAF/NRAS status were analyzed in 14 patients who received ICI treatment.

^b^ PD-L1 evaluation was performed in 12 patients who received ICI treatment.

Gene mutation status was evaluated in 14 patients. The incidences of BRAF and NRAS mutations were observed in no patient (0%) and 4 patients (12.5%), respectively. PD-L1 evaluation was performed on 12 patients. Among them, three patients (9.4%) showed PD-L1 positive, while 9 patients (28.1%) were PD-L1 negative.

### Treatment outcome of all patients

**Figure 2:**
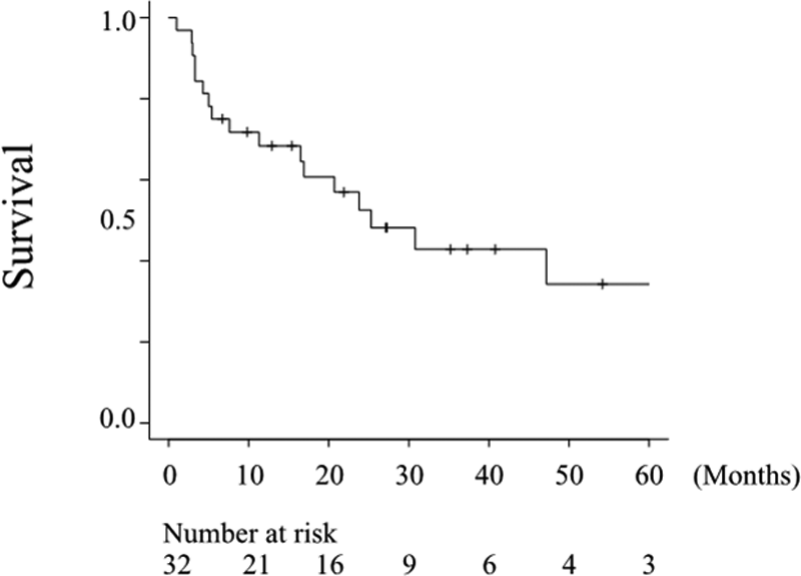
Overall survival of all patients. The median OS after treatment for the advanced disease was 25.3 months (95% CI, 11.3–112.9), and the estimated 5-year OS rate was 34.3%.

The survival analysis of all 32 patients is shown in Figure 2. The median OS of patients with advanced HNMM was 25.3 months (95% CI, 11.3–112.9), and the estimated 1-, 3-, and 5-year OS rates were 68.4%, 42.8%, and 34.3%, respectively.

### Treatment outcomes of patients who received ICI

**Figure 3:**
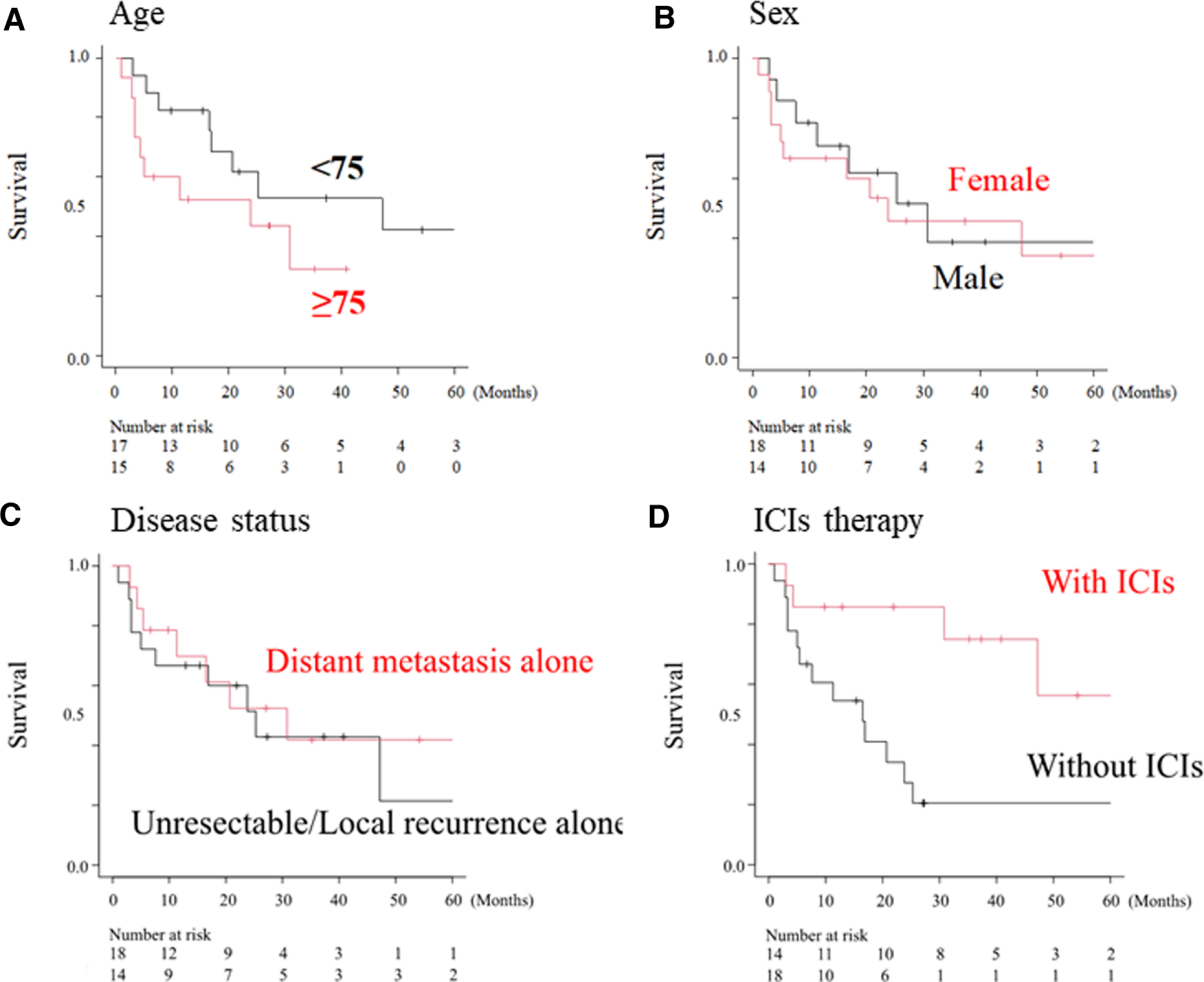
Overall survival curve according to clinical factors. Overall survival according to sex (**A**), age (**B**), disease status (**C**) and ICI treatment (**D**). The median overall survival in patients who received ICI therapy (with ICI) was significantly longer than that in patients without ICIs (without ICI group) (not reached vs. 11.3 months, *p* < 0.01).

Fourteen of the 32 patients received ICI therapy, and CR, PR, SD, and PD were achieved in four, three, two, and five of these patients, respectively (data not shown). Therefore, the ORR and disease control rates were 50% and 64.3%, respectively. The median survival times of patients with or without ICI therapy were not reached (95% CI, 30.8-NA) and 11.3 months (95% CI, 4.7–25.3), respectively (Figure 3). Patients who received ICI therapy showed a significantly better OS than those who did not (*p* = 0.007, Figure 3A).

**Figure 4.**
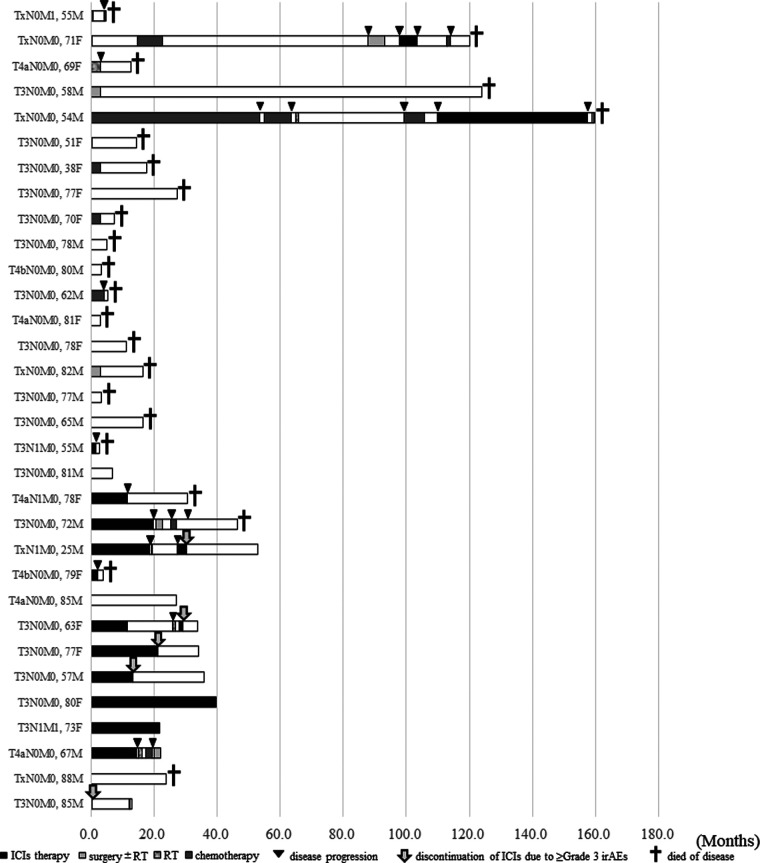
Swimmer plot showing the detailed clinical courses of patients with advanced HNMM (*n* = 32). The data are shown in chronological order of treatment initiation for advanced disease. Black bars; the period of ICIs therapy. Light gray bars; the period of salvage surgery ± following radiotherapy. Striped gray bars; the period of radiotherapy. Dark gray bars; the period of chemotherapy. White bars; off treatment, Black triangles; disease progression. Gray arrow; the onset of irAEs (≥Grade 3) Black cross; Died of disease.

Detailed clinical courses of all 32 patients are shown as swimmer plots in Figure 4. Ten and twenty-two patients were alive and died of the disease, respectively. Among the patients who received ICIs therapy (shown by black bars), the median duration of ICI therapy as the first-line treatment for advanced HNMM was 12.95 months (0.5–47.4 months) (data not shown). Nine patients (64.3%) experienced irAEs (data not shown). During the follow-up period, two patients continued ICI therapy, whereas 12 patients discontinued it due to disease progression (*n* = 7) or ≥grade 3 irAEs (*n* = 5). All five patients who discontinued treatment due to irAEs achieved long survival (9.8–54.2 months) at the end of the follow-up period. Furthermore, four survived after discontinuation without salvage treatment.

### Clinical parameters associated with Os in patients with advanced HNMM

We investigated the clinical parameters associated with 1-year OS in patients with advanced HNMM. As shown in Table 3, univariate analysis revealed that ICI therapy was the only significant factor associated with a better OS. Because all BRAF mutations were negative in all patients, BRAF status was omitted from the univariate analysis.

**Table 3:** Clinical parameters associated with OS in patients with advanced HNMM.

	*n* = 32	1-year overall survival	3-year overall survival	5-year overall survival	*p*-value
**Sex**					
Male	18	66.7%	45.7%	34.3%	0.974
Female	14	70.7%	38.7%	38.7%	
**Age**					
<75	17	82.4%	52.9%	42.4%	0.182
≥75	15	52.5%	29.2%	NA	
**Primary site**					
Sinonasal cavity	26	64.7%	41.5%	20.7%	0.727
Other	6	83.3%	50.0%	50.0%	
**Disease status**					
Unresectable/Local recurrence alone	18	66.7%	42.9%	21.4%	0.957
Distant metastasis alone	14	69.8%	41.9%	41.9%	
**Treatments**					
With ICI	14	85.7%	75.0%	56.2%	**0** **.** **007**
Without ICI	18	54.5%	21.0%	20.5%	
**NRAS status (*n* = 14)**					
Wild	10	90.0%	75.0%	75.0%	0.258
Mutant	4	75.0%	75.0%	NA	
**PD-L1 status (*n* = 12)^a^**					
<5%	9	88.9%	66.7%	NA	0.445
≥5%	3	100.0%	NA	NA	

ICI, immune checkpoint inhibitor; NRAS, neuroblastoma RAS viral oncogene homolog; PD-L1, programmed death-ligand 1.

*Bold: statistically significant.

^a^ PD-L1 evaluation was performed on 12 of advanced HNMM patients.

### Clinical parameters associated with Os after ICI therapy

For the clinical parameters associated with 1-year OS after ICI therapy, univariate analysis revealed that the 1-year OS of patients who achieved CR or PR after ICIs was 100%, which was significantly better than that of patients who showed SD or PD (71.4%, *p* = 0.036) (Table 4).

**Table 4:** Univariate analysis of overall survival on patients who received immune checkpoint inhibitors therapy.

	*n* = 14	1-year verall survival	*p*-value
**Sex**			
Male	7	85.7%	0.514
Female	7	85.7%	
**Age**			
<75	9	88.9%	0.291
≥75	5	80.0%	
**Primary site**			
Sinonasal cavity	11	90.9%	0.701
Other	3	66.7%	
**Disease status**			
Unresectable/Local recurrence	7	100.0%	0.169
Distant metastasis	7	71.4%	
**NRAS status**			
Wild	10	90.0%	0.258
Mutant	4	75.0%	
**PD-L1 status^a^ (*n* = 10)**			
<5%	7	85.7%	0.411
≥5%	3	100.0%	
**Overall response rate**			
CR or PR	7	100.0%	**0** **.** **036**
SD or PD	7	71.4%	
**Disease control rate**			
CR, PR or SD	9	100.0%	0.105
PD	5	60.0%	
**The onset of irAE**			
With irAE	9	100.0%	0.307
Without irAE	5	60.0%	
**Discontinuation of ICIs therapy due to severe irAE**			
Discontinuation	5	100.0%	0.147
Not discontinuation	9	77.8%	

NRAS, neuroblastoma RAS viral oncogene homolog; PD-L1, programmed death-ligand 1; CR, complete response; PR, partial response; SD, stable disease; PD, progressive disease; irAE, immune-related adverse event; ICI, immune checkpoint inhibitor.

**Bold: statistically significant.**

^a^PD-L1 evaluation was performed in 10 patients who received ICI treatment.

## Discussion

Locoregional recurrence and distant metastasis often occur even after intensive treatments, such as surgery in combination with postoperative radiotherapy (24) and carbon-ion radiation (25), suggesting that the development of more effective treatment strategies for recurrent/metastatic HNMM is desirable. Indeed, only four of the 36 patients in this study achieved no recurrence or metastasis after the initial definitive treatment (Figure 1). Therefore, we focused on the treatment outcomes of HNMM patients with advanced disease, including five patients with unresectable or distant metastasis at the initial diagnosis and 27 patients with recurrence/metastasis after initial treatments including surgery plus postoperative radiotherapy and carbon-ion radiotherapy (Figure 1).

Among the 32 HNMM patients with advanced disease, 14 were treated with ICI. The remaining 18 patients received treatments other than ICI: two patients underwent surgery, four received RT, one received chemotherapy, and 11 received the best supportive care. The median OS of the 32 patients was 25.3 months and the estimated rates of 1-, 3-, and 5-year OS were 68.4%, 42.8%, and 34.3%, respectively (Figure 2). López et al. reported that the 5-year OS of HNMM was less than 30% in most of the cited articles that included non-advanced diseases (10). As Although the present study enrolled only patients with advanced HNMM, the survival rate was comparable to or better than that in López's review (10). This could be partly because 14 patients who received ICI treatment were enrolled in our study, which was approved in 2014 in Japan. Moreover, the univariate analysis demonstrated that ICI therapy was the only factor that significantly improved OS in patients with advanced HNMM (Table 3 and Figure 3). According to a systematic review collecting 52 papers that reported the significance of ICI for MM, immunotherapy improved overall survivals (26). These findings may suggest two key-points for the establishment of optimal treatment strategy for HNMM. First, recurrent-free control of HNMM only with high-intensity initial treatment is extremely difficult. Second, ICI is an essential treatment option for recurrent/metastatic HNMM (advanced HNMM).

Regarding the response of 14 patients who received ICI therapy, CR, PR, SD, and PD were achieved in four, three, two, and five patients, respectively. Thus, the ORR and disease control rates were 50% and 64.3%, respectively. The median survival time of patients who received ICI therapy was not reached (Figure 3), and the 1-year OS rate of patients who received ICI was 85.7% (Table 3). Although no reports focusing on HNMM are available, a few studies have reported the efficacy of ICI therapy for MM of mixed primary sites, including the gastrointestinal and urogenital tracts. The ORR of anti- programmed death-1 monotherapy was 15%–30%, and the ORR of combined administration of anti-CTL antigen 4 and anti-programmed death-1 agents was 28%–37% (19–21, 27, 28). Since the ORR exclusive to HNMM in the present study was 50%, MM derived from the head and neck regions may be suitable targets for ICI therapy among MM of various primary sites.

Regarding the clinical parameters associated with OS after ICI therapy, CR/PR to ICI therapy was associated with a better 1-year OS (Table 4). Moreover, analysis of detailed clinical courses in patients who received ICI therapy revealed that patients who discontinued ICI therapy due to severe irAEs achieved long-term survival (Figure 4). Surprisingly, four of the five patients achieved a durable response for more than 1 year without salvage therapy (Figure 4). The presence of irAEs has been reported to be a favorable prognostic factor for various types of cancer (29–31). Patients with severe irAEs that resulted in discontinuation of ICI therapy could achieve a durable response without additional treatment (32, 33). Our results did not reach a significant difference; however, all of these findings suggest that the CR/PR to ICI therapy and the discontinuation of ICIs due to severe irAEs may be favorable prognostic factors in the treatment of advanced HNMM.

BRAF mutation is the most common mutation in CM (35%–50%) (34). The efficacy of the combination of BRAF and mitogen-activated protein kinase/extracellular signal-regulated kinase kinase inhibitors has been reported in patients with BRAF-mutated metastatic melanoma (35). However, BRAF mutations have been observed in only 6% of MM cases (34). Therefore, to search for therapeutic targets for MM, MM-specific genetic features different from those of CM should be explored. These include the NF1, NRAS, and C-kit mutations observed in 14%, 8%, and 13% of MM cases, respectively, all of which are more common than BRAF (34). NRAS and BRAF are oncogenes that constitute the mitogen-activated protein kinase pathways. The efficacy of extracellular signal-regulated kinase kinase inhibitors in NRAS-mutated advanced melanoma has been reported recently (36, 37), and a favorable response to ICI therapy in NRAS-mutated melanoma (38) has also been reported, suggesting the potential role of NRAS mutations as a novel therapeutic target in HNMM. In the present study, no BRAF mutation was observed, and NRAS mutations were confirmed in four of the 14 patients (29%). The NRAS-positive rate seemed to be higher than that in previous reports (34); however, treatment outcomes were not different between patients with and without NRAS mutations (Table 1), suggesting that NRAS mutations play only a minor role in predicting favorable outcomes of ICI therapy for advanced HNMM. However, because we analyzed BRAF/NRAS mutations in only a small number of patients, the results would not be conclusive.

This study had several limitations. First, the study was retrospective in nature, and the sample size was small because of the rarity of HNMM. Second, we analyzed BRAF/NRAS mutations in a small number of patients who received ICI therapy. Future multicenter, large-cohort studies are needed to establish the optimal treatment strategy for HNMM, including ICI therapy, and to detect novel therapeutic targets through genomic analysis.

## Conclusion

The median OS of the 32 patients with advanced HNMM, which was defined as unresectable/distant metastasis at the initial diagnosis or recurrence/metastasis after initial treatment, was 25.3 months. ICI treatment was the only factor associated with a better OS in patients with advanced HNMM. The ORR of the 14 patients who received ICI therapy was 50%, and the 1-year OS was 85.7%. Among patients who received ICI treatment, the ORR was a favorable predictor of OS. Moreover, patients who discontinued ICI therapy owing to severe irAEs achieved a durable response without salvage treatment, which might be an alternative prognostic factor.

## Data Availability

The original contributions presented in the study are included in the article/supplementary material, further inquiries can be directed to the corresponding author/s.
